# Supportive care and antiviral treatments in primary herpetic gingivostomatitis: a systematic review

**DOI:** 10.1007/s00784-023-05250-5

**Published:** 2023-09-21

**Authors:** Noemi Coppola, Tiziana Cantile, Daniela Adamo, Federica Canfora, Stefania Baldares, Francesco Riccitiello, Gennaro Musella, Michele Davide D. Mignogna, Stefania Leuci

**Affiliations:** 1 Oral Medicine Unit, Department of Neuroscience, Reproductive and Odontostomatological Sciences, University of Naples Federico II, Naples, Italy; 2 Pediatric Dentistry, Department of Neuroscience, Reproductive and Odontostomatological Sciences, School of Medicine, University of Naples Federico II, Via Sergio Pansini 5, 80131 Naples, Italy; 3 Department of Medicine, Surgery and Dentistry Scuola Medica Salernitana, University of Salerno, Baronissi, Italy

**Keywords:** Gingivostomatitis, Herpes simplex virus, Therapy, Primary herpetic gingivostomatitis, Antiviral therapy

## Abstract

**Objectives:**

Herpes simplex virus 1 (HSV-1) is the main pathogen responsible for herpes infections. In 13–30% of the cases, primary HSV-1 leads to the primary herpetic gingivostomatitis (PHGS), often a self-limiting infection; however, it can limit the ability to drink/eat with, sometimes, the need for hospitalization. Multiple therapeutic methods have been proposed. This systematic review aims to collect and critically appraise the available evidence about the clinical management of PHGS.

**Materials and methods:**

Literature search including three databases (PubMed, Scopus, Embase), study design, and data analysis were performed following PRISMA guidelines, according to the PICO tool (PROSPERO n° CRD42023391386). Risk of bias was assessed with RoB 2 and ROBINS-I.

**Results:**

Five studies on a total of 364 patients (average age: 7.6 years) were identified. The treatment regimens were summarized in acyclovir; acyclovir + honey; fluids and analgesic; maalox + diphenhydramine; lidocaine; chlorhexidine (CHX); CHX + ialuronic acid; CHX + Mucosyte®; antimicrobial photodynamic therapy (aPDT); topical antiviral; topical antiviral + aPDT; and others.

**Conclusions:**

Although PHGS is a disease with a high worldwide prevalence, the lack of consensus about therapeutic management indicates gaps in existing evidence. Most of the proposed treatment consists in symptomatic drugs with empiric regimens which are ineffective for the viral replication. The main limit to realize randomized clinical trial is due to the rapid onset and remission of the disease. In fact, the diagnostic delay, estimated in 72 h, decreases the effectiveness of any antiviral drugs.

**Clinical relevance:**

Out of the five studies included in this systematic review, only one was able to provide some weak evidence that ACV is an effective treatment, improving healing of oral lesions and reducing duration of symptoms.

**Supplementary Information:**

The online version contains supplementary material available at 10.1007/s00784-023-05250-5.

## Introduction

Herpes viruses are double-stranded DNA viruses, belonging to the Herpesviridae family that includes over 200 members. They have a characteristic four-layered structure: a core containing the large, double-stranded DNA genome; an icosapentahedral capsid; an amorphous protein coat, called the tegument; and a glycoprotein-bearing lipid bilayer envelope [1, 2].

Among herpes viruses, eight are capable of infecting humans, including herpes simplex virus (HSV) 1 and 2, varicella-zoster virus (VZV), Epstein-Barr virus (EBV), cytomegalovirus (CMV), human herpesvirus 6 (HHV-6), human herpesvirus 7 (HHV-7), and Kaposi’s sarcoma virus (KSHV) [2].

A hallmark of herpesviruses is that they effectively infect many cell types, but a specific tissue or cell type is targeted to establish a reactivatable, life-long latent infection. At intervals, the virus can be reactivated from its latent state, causing a clinical recurrence of the disease [1]. The stimuli that trigger reactivation in humans can be heat, cold, trauma, fever, stress, and changes in host’s immune defense status [1].

Among herpes viruses, herpes simplex virus 1 (HSV-1) is the main pathogen responsible for infections worldwide [3]. The transmission of the HSV-1 occurs through direct contact with the lesion or with biological fluids containing the virus, such as saliva and genital fluids. The percentage of viral shedding varies according to the host’s immune status and the stage of the disease; in fact, it is greater in immunocompromised patients and in the prodromal phases of the primary disease [1]. HSV-1 is a pathogen characterized by high infectivity, and most people come into contact with the virus in their lifetime and harbor the virus in a latent form; hence, there is a high spread of the virus with no seasonal variation.

According to the latest available estimate, dating back to 2016 and provided by WHO, 3.7 billion people under the age of 50, or 67% of the population, had HSV-1 infection (oral or genital) (https://www.who.int/news-room/fact-sheets/detail/herpes-simplex-virus). These data are underestimated, because the infection is often asymptomatic or not recognized. HSV-1 gives rise to painful vesicular lesions in the oral and perioral area; more rarely it can cause genital manifestations [2]. The natural history of HSV-1 consists in primary infection, latent infection, and reactivation. During primary infection, viral replication and lytic activity of the virus are minimal, generally not capable of causing clinical manifestations or cause symptoms so mild that patients are unaware of the illness [4, 5]. However, in 13–30% of children and adolescents, primary HSV-1 is symptomatic, and the most common specific clinical manifestation is the primary herpetic gingivostomatitis (PHGS) [6]. Two age peaks of onset of PHGS are recognized: children between 6 months and 5 years old and young adult [7]. Elderly people can also develop PHGS, but with a milder clinical phenotype than younger patients [8]. The incubation period ranges from 2 to 15 days, and, during this phase, prodromal signs may appear, such as fever, asthenia, myalgia, nausea, loss of appetite, irritability, and headache. The specific manifestations of the PHGC arise 1–3 days after the prodromal phase [9]. Clinical appearance of the disease consists in 1–2 mm vesicles, typically in cluster morphology, that break down rapidly, causing painful, irregular erosive lesions with yellowish gray pseudomembrane and perilesional erythema and heal gradually in 10–14 days, without scarring. The most common sites of PHGS are gingiva, labial mucosa, and palate; however, involvement of the tongue and buccal mucosa is also described [10]. Other clinical features include gingival swelling and edema, covered tongue, and halitosis. The oral mucosal and gingival features are usually accompanied to pyrexia, lethargy, and hypersalivation [9].

Less frequently, extraoral manifestations are reported with pharyngeal, nasal, and ocular involvement, submandibular or cervical lymphadenopathy, and initially macular and later purpuric cutaneous rash [11, 12]. After the primary infection, the virus becomes latent in the trigeminal ganglion, but it can be readily reactivated, causing recurrent infections which are generally less severe than the primary infection [1].

Diagnosis is usually clinical, based on the patient’s history and physical examination. Also, it may be confirmed through laboratory tests: serological assays (anti-HSV IgM and IgG), the Tzanck test, and immunofluorescence, but the culture of viral isolates is still considered to be the gold standard [7].

PHGS must be differentiated from other ulcerative diseases, such as herpetiform recurrent aphthous stomatitis, Coxsackie-like virus infection, infectious mononucleosis, erythema multiforme minor, acute necrotizing ulcerative gingivitis, and varicella-zoster virus infection [9].

PHGS is often a self-limiting infection that resolves in 10–14 days; however, serious complications including erythema multiforme, aseptic meningitis, and encephalitis can arise [13]. Furthermore, especially in children, the painful oral lesions limit the ability to drink and eat, causing dehydration with the need for hospitalization in some cases. Also, hospitalization should be considered for immunocompromised children [11].

So, PHGS can cause severe discomfort and lead to reduced quality of life. To date, a wide range of therapeutic methods have been proposed, ranging from supportive treatment, including hydration and pain management to antiviral treatment (e.g., oral acyclovir administered within the first 72 h of disease onset) [14]. However, the lack of consensus indicates gaps in existing evidence. To date, there are no other systematic reviews on this topic. Only a previous systematic review evaluated the effectiveness of systemic acyclovir for PHGS, but it did not include other therapeutic approaches [14].

To bridge this gap, this systematic review aims to collect and critically appraise all the available evidence about the therapeutic strategies proposed for the management of PHGS.

## Materials and methods

The systematic review was designed and conducted according to the Preferred Reporting Items for Systematic Reviews and Meta-Analyses (PRISMA) statement [15]. The study protocol was recorded in the International Prospective Register of Systematic Reviews (PROSPERO) (ID: CRD42023391386).

### Eligibility criteria

The general eligibility criteria for study participation were defined by the “PICO” strategy: “Population”: immunocompetent children and young adults, either gender, any race and ethnicity, with a diagnosis of primary herpetic gingivostomatitis. The diagnosis had been confirmed by clinical history and laboratory investigation; “Intervention”: any systemic, topical, or physical therapy for the treatment of PHGS (the intervention could be either a single intervention or a combination of interventions); “Comparison”: placebo, no intervention, or another active intervention; “Outcomes”: (a) time of healing of oral and extraoral lesions, drooling, eating and drinking difficulties, and fever; (b) severity of oral lesions (change in the number of lesions from the time of administration of the intervention); (c) eating and drinking ability; (d) pain, assessed by a validated pain scale; (e) viral shedding; (f) HSV quantification; (g) biomarkers evaluation.

### Inclusion criteria

Randomized trials which assess the effect of any type of therapy for the treatment of PHGS; non-randomized intervention studies and controlled before after studies; observational studies (including prospective and retrospective cohort and case–control studies, cross-sectional studies).

### Exclusion criteria

Letter to editors, case report or case series, systematic reviews; duplicate studies and/or studies not reporting results after the end of the research; preclinical studies; papers presented at scientific events; articles that had no original data.

### Search strategy

The final search was performed on 1 February 2022 and included the following electronic databases, with no date restrictions: PubMed, Embase, and Scopus. The search terms included “herpetic gingivostomatitis,” “therapy,” “treatment,” and “management.” The elected search terms were combined with Boolean operators for detailed electronic search: “herpetic gingivostomatitis” AND “therapy”; “herpetic gingivostomatitis” AND “treatment”; and “herpetic gingivostomatitis” AND “management.”

### Study selection

Publications in English language or those which had English language translation were included in the analysis. The duplicate articles were removed, and the bibliographies of the pertinent reviews were manually searched for additional references. Screening of titles and abstracts resulting from the initial electronic searches was performed by two independent reviewers (TC and NC).

Full copies of all relevant and potentially relevant studies, appearing to meet the inclusion criteria, or those reporting insufficient data in the title and abstract to make a clear decision, were screened. The full text papers were assessed independently by TC and NC and any disagreement on the eligibility of included studies was resolved through discussion and consensus or, if necessary, through a third reviewer (SL), who acted as an arbiter.

**Fig. 1:**
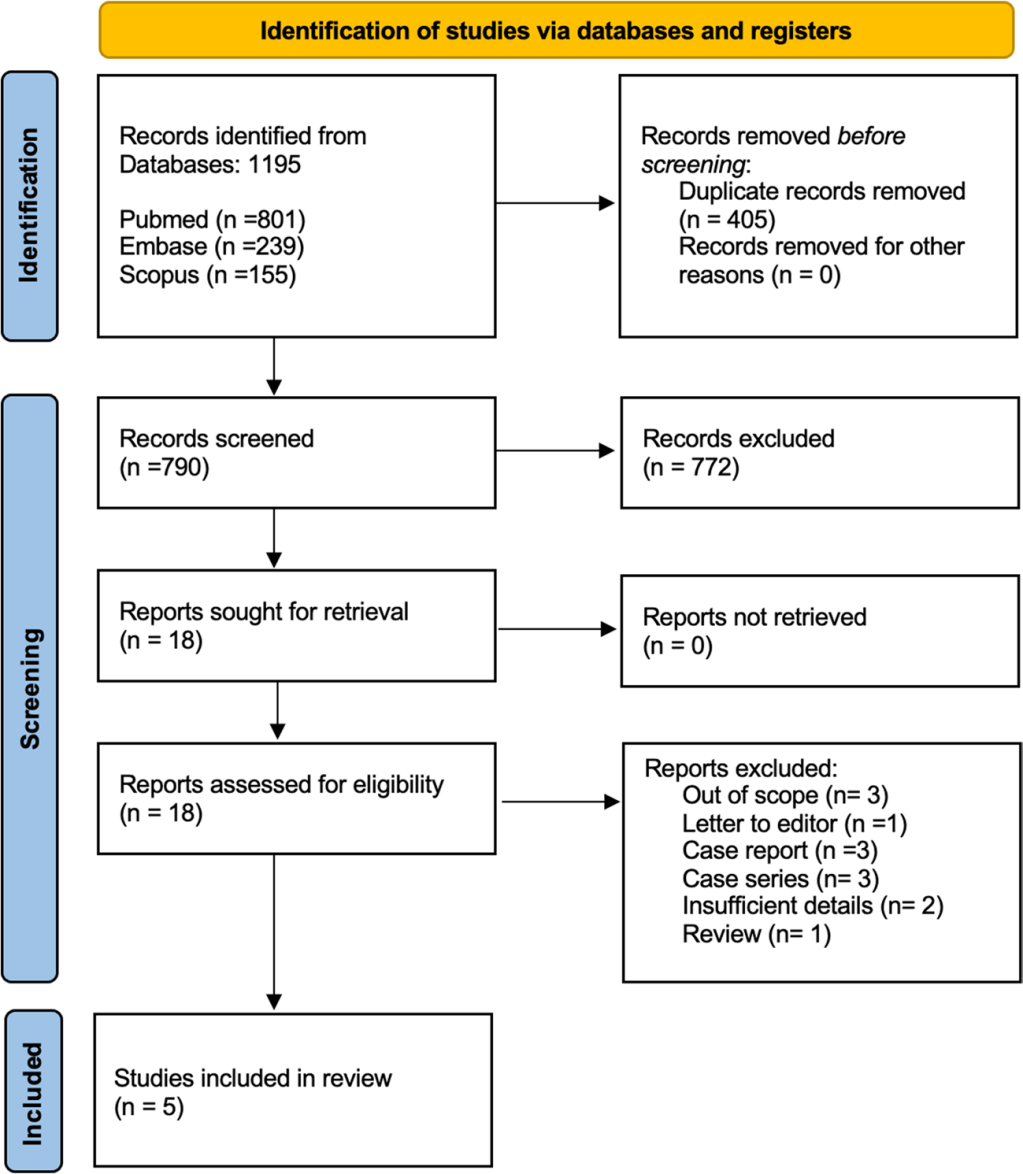
PRISMA flowchart

All irrelevant records were excluded and the reasons for their exclusion were noted. The screening and selection process has been described in a PRISMA flowchart (Fig. 1).

### Data extraction

At least two of the three reviewers (TC, NC, SL) independently extracted data using a standardized predefined data extraction form. Agreement between authors was determined by using Cohen’s kappa value (*K*-statistic: 0.447; standard error: 0.133). The extracted data included the general characteristics of the articles (study design, year published, authors, geographic location), variables relating to study methodology, population, intervention, comparators, and outcomes. Discrepancies were resolved through discussion or through a fourth reviewer (AB).

### Assessment of risk of bias and quality of studies

The risk of bias assessment for each study was done independently and in duplicate by two authors (TC and NC or AB) using either version 2 of the Cochrane risk-of-bias tool (RoB 2), a framework for assessing the risk of bias in a single estimate of an intervention effect reported from a randomized trial, or the Cochrane risk-of-bias tool (ROBINS-I), a tool for evaluating risk of bias in estimates of the comparative effectiveness of interventions from studies that did not use randomization to allocate units to comparison groups [15].

Disagreements were resolved by discussion, with the involvement of a third author (SL), if necessary.

### Data and statistical analysis

From the list of eligible articles, the research team extracted the following data: publication and study characteristics (study design, year published, authors, geographic location), study population characteristics (age, sample size), therapy, duration of follow-ups, and outcomes.

Since meta-analysis was not possible, a narrative summary of the results from individual studies was provided.

## Results

### Study selection

A total of 5 studies on a total of 364 patients (average age: 7.6 years) were identified for inclusion in the review.

The search provided a total of 1195 citations. After adjusting for duplicates, 790 remained. Of these, 772 studies were discarded because, after reviewing titles and abstracts, it appeared that these papers clearly did not meet the criteria. The full text of the remaining 18 citations was examined in more detail. It appeared that 13 studies did not meet the inclusion criteria as described. Five studies met the inclusion criteria and were included in the systematic review. No additional studies that met the criteria for inclusion were identified by checking the references of located, relevant papers and searching for studies that have cited these papers. No unpublished relevant studies were obtained.

### Study characteristics

Characteristics of the included studies have been summarized in Table 1.

**Table 1:**
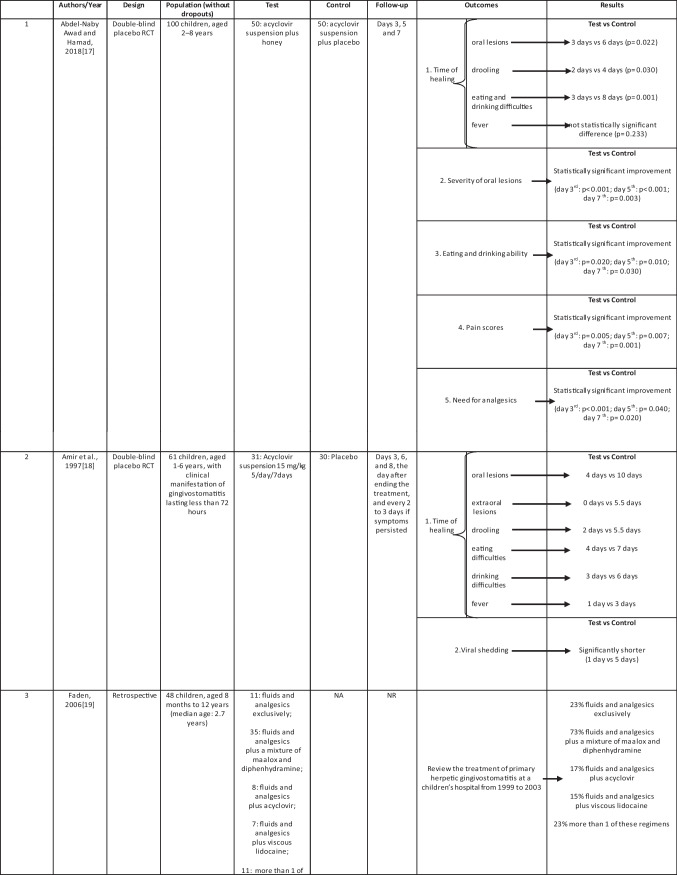

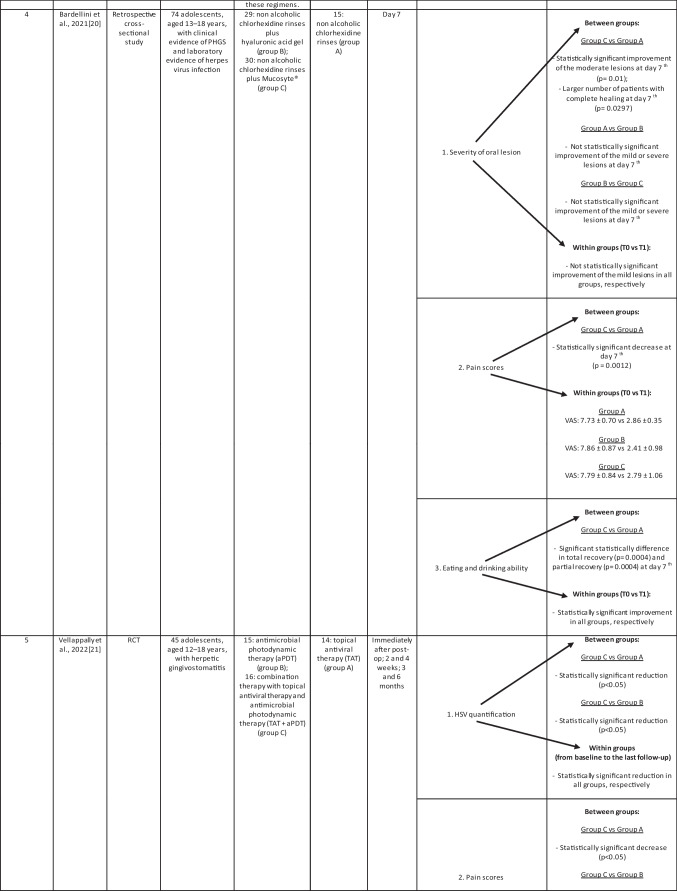

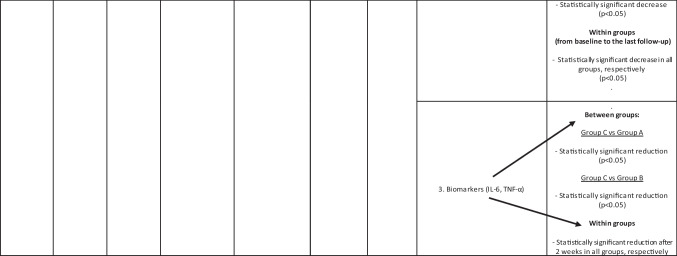
Therapeutic strategies of primary herpetic gingivostomatitis: a systematic review

No comparable therapies were reported in the included literature; therefore, a narrative review was performed.

All included studies enrolled patients with first clinical manifestation of herpetic gingivostomatitis, but, while 3 studies (1, 2, and 3) interested pediatric subjects (8 months–12 years), 2 (4 and 5) included adolescents (12–18 years).

All studies took place in either hospitals or university hospitals, in 5 different countries.

### Risk of bias in studies

Three of the included studies (1, 2, and 5) were randomized clinical trials, while 2 (3 and 4) were retrospective. Overall risk of bias, assessed with RoB 2, was low for 1 and 2, while some concern was determined for 5; overall risk of bias, assessed with ROBINS-I, for studies 3 and 4 was serious and moderate, respectively (Tables 2 and 3).

**Table 2:** Risk of bias—assessment using RoB 2 tool

Studies included in the review	D1	D2	D3	D4	D5	Overall
Study 1	+	+	+	+	+	+
Study 2	+	+	+	+	+	+
Study 5	+	−	+	+	+	−

Judgment:

+ low risk

X high risk

− some concerns

Domains:

D1: bias arising from the randomization process

D2: bias due to deviations from intended intervention

D3: bias due to missing outcome data

D4: bias in measurement of the outcome

D5: bias in selection of the reported result

**Table 3:** Risk of bias—assessment using ROBINS-I tool

Studies included in the review	D1	D2	D3	D4	D5	D6	D7	Overall
Study 3	X	−	−	+	X	−	−	X
Study 4	−	−	−	+	+	+	+	−

Judgment:

! critical

X serious

− moderate

+ low

? no information

Domains:

D1: bias due to confounding

D2: bias due to selection of participants

D3: bias in classification of interventions

D4: bias due to deviations from intended interventions

D5: bias due to missing data

D6: bias in measurement of outcomes

D7: bias in selection of the reported result

### Results of individual studies

#### Study 1

Tests (honey plus oral ACV) had significantly earlier disappearance of herpetic oral lesions: median 3 days vs. 6 days in controls (oral ACV) (*p* = 0.022); drooling: 2 days vs. 4 days (*p* = 0.030); and eating difficulty: 3 days vs. 8 days (*p* = 0.001). Tests also had significantly improvement in severity of oral lesions, lower pain scores, better eating and drinking ability, and significantly less need for analgesics at 3 time-points of assessment. Fever disappeared in both groups with no statistically significant difference [16].

#### Study 2

Children receiving ACV had oral lesions for a shorter period than children receiving placebo (median 4 vs. 10 days (difference 6 days, 95% confidence interval 4.0 to 8.0)) and earlier disappearance of the following signs and symptoms: fever (1 vs. 3 days (2 days, 0.8 to 3.2)); extraoral lesions (0 vs. 5.5 days (5.5 days, 1.3 to 4.7)); eating difficulties (4 vs. 7 days (3 days, 1.31 to 4.69)); and drinking difficulties (3 vs. 6 days (3 days, 1.1 to 4.9)). Viral shedding was significantly shorter in the group treated with acyclovir (1 vs. 5 days (4 days, 2.9 to 5.1)) [17].

#### Study 3

Topical therapy with maalox and diphenhydramine or viscous lidocaine was administered to 73% and 15% of the patients, respectively, whereas ACV was administered to only 17%. Dosing and administration of topical agents in the treatment of PHGS in preschoolers were problematic. ACV was not being used as often as it could have been [18].

#### Study 4

To regard severity of oral lesions, all therapies showed to be efficient, except for mild lesions that in all groups (group A: non-alcoholic chlorhexidine (CHX) rinses; group B: non-alcoholic chlorhexidine rinses plus hyaluronic acid gel; group C: non-alcoholic chlorhexidine rinses plus Mucosyte®) did not show a statistically significant improvement. Moderate lesions: group C showed a significantly larger number of patients with complete healing of the oral mucosa or with an improvement compared to group A. Mild/severe lesions: no difference was recorded.

To regard pain scores, a statistically significant difference was obtained from T0 to T1 for all groups. Pain scores significantly decreased in group C with respect to those in group A.

To regard eating and drinking ability, all patients totally or partially improved the eating and drinking ability. A significant statistically difference was noted between group A and group C in total recovery and in partial recovery. Group C showed a full improvement of the abilities [19].

#### Study 5

In relation to HSV quantification, this value showed a significant reduction in all the study groups (group A: topical antiviral therapy (TAT); group B: antimicrobial photodynamic therapy (aPDT); group C: combination therapy with TAT + aPDT) during every follow-up. Group C reported a statistically higher reduction than group A and group B. The pain scores significantly decreased in all groups. Group C reported a statistically higher decrease than group A and group B. In relation to biomarkers, IL-6 and TNF-α reported statistically significant reduction after 2 weeks. Group C demonstrated a statistically higher reduction between groups and within groups at every time point [20].

### Results of syntheses

ACV was used in combination or alone in 4 of the included studies (1, 2, 3, and 5). When in combination, the other therapies were honey (1), fluids and analgesics (3), and aPDT (5).

When ACV was used alone (study 2), compared to placebo, shortened the duration of all clinical manifestations and the infectivity of affected children.

The combined use of honey and oral ACV (study 1) produced a more favorable outcome (i.e., earlier disappearance of herpetic oral lesions, of drooling, and of eating difficulty; lower pain scores; better eating and drinking ability; significantly less need for analgesics) than acyclovir alone in children with PHGS.

ACV therapy in conjunction with aPDT (study 5) helped in reducing the pain scores and pro-inflammatory cytokine levels in herpetic gingivostomatitis among children.

One study (4) used non-alcoholic chlorhexidine alone or in combination with other substances (i.e., hyaluronic acid or verbascoside and sodium hyaluronate (Mucosyte®)).

When non-alcoholic chlorhexidine was used in combination with Mucosyte®, a significant improvement of the pain scoring and lesions’ severity was noted in comparison to non-alcoholic chlorhexidine alone and non-alcoholic chlorhexidine plus hyaluronic acid, respectively.

The study 3 reported the use of fluids and analgesics alone or in combination with viscous lidocaine and a mixture of maalox and diphenhydramine, but authors did not provide any comparison among the described therapies.

Finally, the studies (4 and 5) that included adolescent patients involved topical therapies and/or non-pharmacological treatments, while the studies (1, 2, and 3) that interested pediatric subjects were focused on ACV, except for study 3 which reported mixed therapies.

### Outcomes measures

Parameters adopted for assessing outcomes of therapies varied greatly between included studies. The most common were eating and drinking abilities/difficulties, as well as severity and duration of oral lesions, pain scores, and fever. Only one study (5) used laboratory tests.

For the outcomes common to at least two studies, the results can be summarized as follows:

- Time of healing of oral lesions: 3 days, 4 days, 6 days, and 10 days for ACV plus honey (study 1), ACV suspension (study 2), ACV suspension plus placebo (study 1), and placebo alone (study 2), respectively.
- Remission of drooling: 2 days, 2 days, 4 days, and 5.5 days for ACV plus honey (study 1), ACV suspension (study 2), ACV suspension plus placebo (study 1), and placebo alone (study 2), respectively.
- Remission of eating difficulties: 3 day, 4 days, 7 days, and 8 days for ACV plus honey (study 1), ACV suspension (study 2), placebo alone (study 2), and ACV suspension plus placebo (study 1), respectively.
- Remission of drinking difficulties: 3 days, 3 days, 6 days, and 8 days for ACV plus honey (study 1), ACV suspension (study 2), placebo alone (study 2), and ACV suspension plus placebo (study 1), respectively.
- Pain scores: a statistically significant reduction was reported for all tests (ACV plus honey; non-alcoholic chlorhexidine rinses plus Mucosyte®; TAT + aPDT) (studies 1, 4, 5) compared to control (ACV plus placebo; non-alcoholic chlorhexidine rinses; TAT) (studies 1, 4, 5).

Finally, also time-points for re-evaluation were not homogeneous between the included studies, but the majority of studies (1, 2, and 4) set the final visit approximately 7 days after the beginning of the therapies. One study (3) did not report the time of follow-ups, while one study (5) examined the patients immediately after post-op and, then, after 2 and 4 weeks, 3 and 6 months.

## Discussion

Despite the high incidence and burden of the PHGS, this systematic review highlights that there is little scientific data on the treatment of the disease. In the 5 included studies, the following therapeutic approaches have been reported: ACV in combination or alone, non-alcoholic chlorhexidine alone or in combination with other substances (i.e., hyaluronic acid or verbascoside and sodium hyaluronate (Mucosyte®)), viscous lidocaine, a mixture of maalox and diphenhydramine, and aPDT. ACV is a safe and well-tolerated antiviral approved in 1982 and represents the first-line treatment of HSV infections, included PHGS [21]. It has a powerful inhibitory effect on viral DNA, without affecting the DNA replication of non-infected cells; so, the toxicity of ACV is weak [22]. The main side effects of ACV are headache, malaise, and vomiting. As reported by Amir et al. oral administration of ACV in the treatment of PHGS is effective if administered within 3 days of the onset of infection [17]. As stated above, the main clinical features of PGHS are oral vesiculo-bullous lesions with a severe involvement of the gingiva. However, in absence of a clear cluster arrangement of the vesicular lesions, pathognomonic sign of the herpes infections, it is difficult to diagnose the PGHS. In addition, often the blistering lesions rapidly break, resulting in unspecific erosions. The diagnosis of PHGS is based on clinical evidence and laboratory evidence with positive serology and positive culture results [23]. Considering diagnostic methods, there is often a diagnostic delay due to both the rapid onset of the illness and rapid resolution, and the time required to obtain laboratory test results. So, the diagnosis of PHGS is made more than 72 h from the onset, when the efficacy of ACV decreases. This diagnostic delay may lead to some complications ranging from erythema multiforme (EM) to life-threatening encephalitis [11]. The most common complication is dehydration, due to difficulty eating and drinking, requiring the administration of fluids and, in some cases, hospitalization [24]. Another morbidity of PHGS is EM, a disorder caused by a cell-mediated immune response [25, 26]. The clinical manifestation of the disease encompasses bullae, macules, and papules that involve the oral mucosa and skin eruption [27]. The most reported triggers for EM are infections agents and drugs, within viral agents HSV-1 and HSV-2 being the most commonly reported precipitators of EM [28–30]. Moreover, HSV-1 causes most cases of herpes simplex encephalitis in adults with a reported mortality of 20–30% and neurological sequelae in survivors [31]. Other complications of PHGS are ocular involvement representing the most frequent cause of corneal blindness in the USA [11]. The clinical manifestations of ocular HSV1 encompass inflammatory vesicles and ulcers, dendritic lesions in the corneal epithelium, stromal opacity, and edema [32, 33]. A rare complication of PHGS in immunocompetent is herpetic esophagitis, commonly described in immunocompromised patients [34]. Odynophagia, heart burn, vomiting, malaise, and appetite loss are the main symptoms referred by patients [35]. Herpetic esophagitis may result from direct extension of oropharyngeal infection, including PHGS [11]. Therefore, the rapid diagnosis of PHGS and the initiation of therapy with ACV within 72 h could help the healing of the PGHS and avoid the onset of the described complications. A RCT from 1997 on 61 children affected by PHGS investigated the efficacy of oral ACV on duration of oral lesions, fever, eating and drinking difficulties, and viral shedding [17]. The study showed that patients treated with systemic ACV had oral lesions for a shorter period than in those receiving placebo and earlier disappearance of all associated signs and symptoms. Moreover, oral ACV treatment shortened the infectivity of the affected children [17]. Amir’s study is the only one, among the studies included in this systematic review, to analyze the effect of acyclovir in an RCT [17]. The other included studies evaluate the efficacy of symptomatic therapies, such as honey, non-alcoholic chlorhexidine rinses, hyaluronic acid gel, Mucosyte®, or antimicrobial photodynamic therapy (aPDT). Hence, therapeutic approaches aimed at relieving symptoms, especially pain, associated with PHGS, rather than targeting viral replication arrest, have been described. In fact, a recent RCT, conducted by Vellappally, compared three groups of provision of treatment: group A: topical antiviral therapy, group B: aPDT, and group C: aPDT + adjunctive topical antiviral therapy [20]. Although the three groups reported an improvement in the outcomes analyzed, group C reported improvement in the pain scores, HSV-1 quantification, and levels of the pro-inflammatory cytokines, which was statistically significant in comparison to treatment with topical antiviral therapy and aPDT alone [20]. This demonstrates that photodynamic therapy can improve the therapeutic action of ACV and act as symptomatic even after the phase of active viral replication. However, the use of aPDT alone, a laser-based therapy whose use is consolidated for many other oral diseases [36], does not achieve the same efficacy as when used in association with ACV.

Another agent described by Abdel-Naby Awad as adjunct treatment for PHGS in children is honey [16]. The combination of honey and ACV has shown to be more helpful in decreasing the severity of symptoms than acyclovir alone. Particularly, the study proved that honey and ACV significantly improved pain, eating and drinking ability, and the consumption of painkillers compared to acyclovir alone. These beneficial effects can be ascribed to the biological and therapeutic properties of honey; its biggest benefit is the anti-inflammatory action, due to the presence of flavonoids and phenolic acids [16, 37]. Moreover, honey has antibacterial properties, as it contains enzymes that produce hydrogen peroxide, which can eliminate harmful bacteria. Kassim et al. found that honey is capable to inhibit both gram-positive and gram-negative bacteria and to both aerobes and anaerobes, including *Staphylococcus aureus* and *Pseudomonas*. Honey’s antibacterial properties can also aid in preventing the development of secondary infections [38]. Additionally, honey has wound healing properties, which include the stimulation of tissue growth, enhanced epithelialization, and minimized scar formation. These effects are attributed to honey’s acidity, hydrogen peroxide content, osmotic effect, nutritional and antioxidant contents, stimulation of immunity, and unidentified compounds [39]. Despite its accessibility, affordability, and lack of specific side effects, the highly cariogenic nature of honey should be considered when using it as a therapeutic approach. Consumption of honey can provide a substrate for cariogenic bacteria to proliferate and produce acids that erode tooth surfaces. Furthermore, honey contains organic acids that can contribute to tooth demineralization and inhibit the remineralization of tooth structure [40]. It is critical to highlight that in the study by Abdel-Naby Awad the long-term consequences of honey use in children have not been evaluated, which is a concern when considering honey as a therapeutic alternative [16]. Therefore, the highly cariogenic nature of honey and the lack of long-term evaluations of its effects on children should be carefully weighed when using it as a management strategy.

As stated above, one of the main goals of therapy is to reduce the symptoms associated with PHGS, especially the pain. Bardellini et al. compared the efficacy of non-alcoholic chlorhexidine rinses used alone and used in association with hyaluronic acid gel or with Mucosyte® [19]. Although all therapies showed to be efficient, a significant improvement of the pain scoring and lesions’ severity was reported with the combination of non-alcoholic chlorhexidine rinses and Mucosyte®. This agent is a solution composed by verbascoside, polyvinylpyrrolidone, and sodium hyaluronate with reparative action, adhering to the oral mucosa and forming a protective film [41]. Furthermore, Faden reported a mixture of maalox and diphenhydramine, even in the absence of scientific evidence on the benefit of this or similar mixtures [18]. Also, the dose of the mixture, the method, and the frequency of administration raised several concerns because the intentional or inadvertent ingestion of relatively large amounts of diphenhydramine can be a potential risk for sedation to young infants. These same concerns apply to the use of viscous lidocaine. In addition, the ingestion of viscous lidocaine has been associated with the risk for seizures. Unexpectedly, ACV was used relatively infrequently in the report by Faden [18].

This study has some limitations. First, the studies included in this systematic review did not adopt a standardized research protocol, making it difficult to compare the results of different studies. Also the heterogeneity of the outcomes between the studies limits the comparison between them. Second, caution in the interpretation of the results must be advised due to some methodological limitations, incompleteness of data in the study 3, and the likelihood of bias in respect of outcomes assessment. Third, the clinical applicability of the findings is limited to the treatment of symptoms secondary to PHGS. In fact, most of the proposed treatment consists in symptomatic drugs with empiric regimens which are ineffective for the viral replication. Furthermore, in relation to the ACV-based therapy, generally used as the main drug for PHGS treatment, current evidence on its therapeutic benefits is limited, despite the extensive utilization. Out of the five studies included in this systematic review, only one was able to provide some weak evidence that ACV is an effective treatment. Standardizing protocols through RCTs can warrant consistency in the PHGS therapy, resulting in better outcomes and decreasing the variability of treatment approaches. Therefore, it is essential to design well-structured RCTs in order to establish standardized interventions in the clinical management of PHGS, when a pharmacological therapy is required. Unfortunately, the main limit to realize randomized clinical trial is due to the rapid onset and remission of the disease. In fact, the diagnostic delay, estimated in 72 h, decreases the effectiveness of any antiviral drugs.

Definitively, although PHGS is a disease with a high worldwide prevalence, the lack of consensus about therapeutic management indicates gaps in existing evidence. The studies included in this systematic review led us to affirm that, among the reported therapies in the scientific literature, there is not enough evidence to prefer one therapeutic approach than another. Furthermore, to date, in the majority of the included studies, the therapy of PHGS aims mainly to reduce the discomfort of the patients rather than the viral shedding.

### Supplementary Information

Below is the link to the electronic supplementary material.

Supplementary file1 (DOCX 32 KB)

Supplementary file2 (DOCX 15 KB)

Supplementary file3 (DOCX 19 KB)

## Data Availability

The data that support the findings of this study are available from the corresponding author upon request.
