# Supplementary material for: Supportive care and antiviral treatments in primary herpetic gingivostomatitis: a systematic review
Source: Clin Oral Investig. 2023 Sep 21;27(11):6333–44. doi: 10.1007/s00784-023-05250-5 (PMC10630243; doi:10.1007/s00784-023-05250-5)
Supplement: Supplementary file 2 — Supplementary file2 (DOCX 15 KB) [file 784_2023_5250_MOESM2_ESM.docx]

## PubMed Search strategy - Search conducted on 9 December 2022

**#1** **Search: ((treatment) OR (therapy)) AND (herpetic gingivostomatitis) Sort by: Publication Date**

("therapeutics"[MeSH Terms] OR "therapeutics"[All Fields] OR "treatments"[All Fields] OR "therapy"[MeSH Subheading] OR "therapy"[All Fields] OR "treatment"[All Fields] OR "treatment s"[All Fields] OR ("therapeutics"[MeSH Terms] OR "therapeutics"[All Fields] OR "therapies"[All Fields] OR "therapy"[MeSH Subheading] OR "therapy"[All Fields] OR "therapy s"[All Fields] OR "therapys"[All Fields])) AND ("stomatitis, herpetic"[MeSH Terms] OR ("stomatitis"[All Fields] AND "herpetic"[All Fields]) OR "herpetic stomatitis"[All Fields] OR ("herpetic"[All Fields] AND "gingivostomatitis"[All Fields]) OR "herpetic gingivostomatitis"[All Fields])

- N=672

**#2 Search: (management) AND (herpetic gingivostomatitis) Sort by: Publication Date**

("manage"[All Fields] OR "managed"[All Fields] OR "management s"[All Fields] OR "managements"[All Fields] OR "manager"[All Fields] OR "manager s"[All Fields] OR "managers"[All Fields] OR "manages"[All Fields] OR "managing"[All Fields] OR "managment"[All Fields] OR "organization and administration"[MeSH Terms] OR ("organization"[All Fields] AND "administration"[All Fields]) OR "organization and administration"[All Fields] OR "management"[All Fields] OR "disease management"[MeSH Terms] OR ("disease"[All Fields] AND "management"[All Fields]) OR "disease management"[All Fields]) AND ("stomatitis, herpetic"[MeSH Terms] OR ("stomatitis"[All Fields] AND "herpetic"[All Fields]) OR "herpetic stomatitis"[All Fields] OR ("herpetic"[All Fields] AND "gingivostomatitis"[All Fields]) OR "herpetic gingivostomatitis"[All Fields])

- N=129
