# Supplementary material for: Supportive care and antiviral treatments in primary herpetic gingivostomatitis: a systematic review
Source: Clin Oral Investig. 2023 Sep 21;27(11):6333–44. doi: 10.1007/s00784-023-05250-5 (PMC10630243; doi:10.1007/s00784-023-05250-5)
Supplement: Supplementary file 3 — Supplementary file3 (DOCX 19 KB) [file 784_2023_5250_MOESM3_ESM.docx]

| **Authors** | **Title** | **Journal** | **Year** | **Motivation for exclusion** |
| --- | --- | --- | --- | --- |
| Amir J, Harel L, Smetana Z, Varsano I | The natural history of primary herpes simplex type 1 gingivostomatitis in children | Pediatr Dermatol | 1999 | Insufficient details: no data on therapy |
| Indra G, Maragathavalli G, Deepika R | Analysis of various treatment modalities of herpetic lesions | Int J Res Pharm Sci | 2020 | Insufficient details: it was impossible to extract data for the outcome of interest |
| Chauvin PJ, Ajar AH | Acute herpetic gingivostomatitis in adults: a review of 13 cases, including diagnosis and management | J Can Dent Assoc | 2002 | Case series |
| McCord | Treatment of children for primary acute herpetic gingivostomatitis with lactobacillus in aqueous suspension | Pediatr Dent | 1988 | Case series |
| Partridge M, Poswillo DE | Topical carbenoxolone sodium in the management of herpes simplex infection | Br J Oral Maxillofac Surg | 1984 | Case series |
| Tang SJ, McCrady W, Rawls D | Acute herpes simplex gingivostomatitis and esophagitis (with video). | Gastrointest Endosc | 2011 | Case report |
| Hudson B, Powell C | Towards evidence based medicine for paediatricians. Does oral aciclovir improve clinical outcome in immunocompetent children with primary herpes simplex gingivostomatitis? | Arch Dis Child | 2009 | Case report |
| Poswillo DE, Roberts GJ | Topical carbenoxolone for orofacial herpes simplex infections | Lancet | 1981 | Case report |
| Kimberlin DW | Herpes simplex virus infections in neonates and early childhood | Semin Pediatr Infect Dis | 2005 | Review |
| Kolenko YG, Timokhina TO, Khrol NS, Kononova OV, Lynovytska OV | Effectіveness of laser therapy іn complex treatment of herpetic stomatitis | Wiad Lek | 2021 | Out of scope: the paper was focused on secondary herpes labialis and not on primary herpetic gingivostomatitis |
| Tod M, Lokiec F, Bidault R, De Bony F, Petitjean O, Aujard Y | Pharmacokinetics of oral acyclovir in neonates and in infants: a population analysis | Antimicrob Agents Chemother | 2001 | Out of scope: the paper was focused on pharmacokinetics of oral acyclovir |
| Sullender WM, Arvin AM, Diaz PS, Connor JD, Straube R, Dankner W, Levin MJ, Weller S, Blum MR, Chapman S | Pharmacokinetics of acyclovir suspension in infants and children | Antimicrob Agents Chemother | 1987 | Out of scope: the paper was focused on pharmacokinetics of acyclovir suspension |
| Laudenbach JM | Oral medicine update: infectious oral lesions | J Calif Dent Assoc | 2013 | Letter to editor |

**Studies excluded in the final decision-making process.**

**References**

Amir J, Harel L, Smetana Z, Varsano I. The natural history of primary herpes simplex type 1 gingivostomatitis in children. Pediatr Dermatol. 1999 Jul-Aug;16(4):259-63. doi: 10.1046/j.1525-1470.1999.00072.x.

Indra G, Maragathavalli G, Deepika R. Analysis of various treatment modalities of herpetic lesions. Maragathavalli G et al., Int. J. Res. Pharm. Sci., 2020, 11 (SPL3), 1830-1836.

Chauvin PJ, Ajar AH. Acute herpetic gingivostomatitis in adults: a review of 13 cases, including diagnosis and management. J Can Dent Assoc. 2002 Apr;68(4):247-51.

McCord WF. Treatment of children for primary acute herpetic gingivostomatitis with lactobacillus in aqueous suspension. Pediatr Dent. 1988 Dec;10(4):307-8.

Partridge M, Poswillo DE. Topical carbenoxolone sodium in the management of herpes simplex infection. Br J Oral Maxillofac Surg. 1984 Apr;22(2):138-45. doi: 10.1016/0266-4356(84)90026-3.

Tang SJ, McCrady W, Rawls D. Acute herpes simplex gingivostomatitis and esophagitis (with video). Gastrointest Endosc. 2011 Jul;74(1):195-6; discussion 196. doi: 10.1016/j.gie.2011.02.021.

Hudson B, Powell C. Towards evidence based medicine for paediatricians. Does oral aciclovir improve clinical outcome in immunocompetent children with primary herpes simplex gingivostomatitis? Arch Dis Child. 2009 Feb;94(2):165-7. doi: 10.1136/adc.2008.145482.

Poswillo DE, Roberts GJ. Topical carbenoxolone for orofacial herpes simplex infections. Lancet. 1981 Jul 18;2(8238):143-4. doi: 10.1016/s0140-6736(81)90318-4.

Kimberlin DW. Herpes simplex virus infections in neonates and early childhood. Semin Pediatr Infect Dis. 2005 Oct;16(4):271-81. doi: 10.1053/j.spid.2005.06.007.

Kolenko YG, Timokhina TO, Khrol NS, Kononova OV, Lynovytska OV. Effectіveness of laser therapy іn complex treatment of herpetic stomatitis. Wiad Lek. 2021;74(6):1331-1335.

Tod M, Lokiec F, Bidault R, De Bony F, Petitjean O, Aujard Y. Pharmacokinetics of oral acyclovir in neonates and in infants: a population analysis. Antimicrob Agents Chemother. 2001 Jan;45(1):150-7. doi: 10.1128/AAC.45.1.150-157.2001.

Sullender WM, Arvin AM, Diaz PS, Connor JD, Straube R, Dankner W, Levin MJ, Weller S, Blum MR, Chapman S. Pharmacokinetics of acyclovir suspension in infants and children. Antimicrob Agents Chemother. 1987 Nov;31(11):1722-6. doi: 10.1128/AAC.31.11.1722.

Laudenbach JM. Oral medicine update: infectious oral lesions. J Calif Dent Assoc. 2013 Apr;41(4):257-8.
